# Supplementary material for: Ligand Binding Properties of Odorant-Binding Protein OBP5 from Mus musculus
Source: Biology (Basel). 2022 Dec 20;12(1):2. doi: 10.3390/biology12010002 (PMC9855133; doi:10.3390/biology12010002)
Supplement: Supplementary file 1 [file biology-12-00002-s001.zip › biology-2064779-supplementary.pdf]

Ligand binding properties of odorant-binding protein OBP5 from *Mus musculus*

Lucie Moitrier [1], Christine Belloir [2], Maxence Lalis [3], Yanxia Hou [4], Jérémie Topin [5,*] and Briand Loïc [6,*]

Supplementary

```
        10         20         30         40         50         60
MRGCHHHHHH TDPAMEGPWK TVAIAADRVD KIERGGELRI YCRSLTCEKE CKEMKVTFYV

        70         80         90        100        110        120
NENGQCSLTT ITGYLQEDGK TYKTQFQGNN RYKLVDESPE NLTFYSENVD RADRKTKLLF

       130        140        150        160
ILGHGPLTSE QKEKFAELAE EKGIPAGNIR EVLITDYCPE
```

Figure S1. Amino acid sequence of recombinantly expressed mOBP5. The mOBP5 sequence (Ala14-Glu160) is shown with a green background. Numbers refer to amino acid residues of mOBP5 (signal peptide: 1–16). The His-Tag sequence is shown in yellow. This tag was used for purification and immunodetection of the protein.

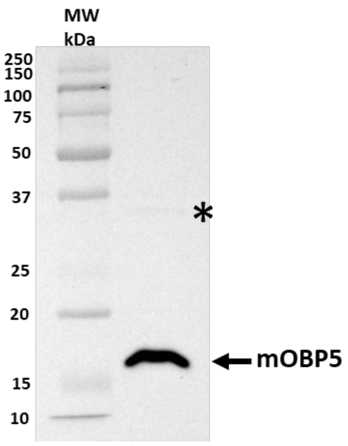

Figure S2. Western blot analysis of mOBP5 expressed in *E. coli* using the pQE-31 vector. The purified protein from the gel filtration was separated by SDS-PAGE followed by western blotting using mouse anti-HIS primary antibody and goat anti-mouse horseradish peroxidase conjugated secondary antibody. The arrow indicates position of purified mOBP5. The asterisk indicates a band probably corresponding to a SDS-resistant dimer of mOBP5.

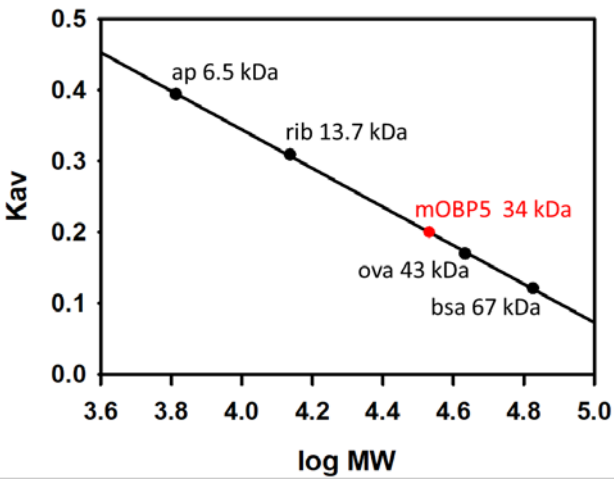

Figure ~~S3~~S2: Calibration curve used to estimate the native molecular weight of purified mOBP5. The calibration curve for the Superdex 75 column (GE Healthcare) was established with bovine serum albumin~~e~~ (bsa, 67 kDa), ovalbumin (ova, 43 kDa), carbonic ribonuclease (rib, 13.7 kDa) and aprotinin (ap, 6.5 kDa). The estimated molecular mass of mOBP5 is 34 kDa.

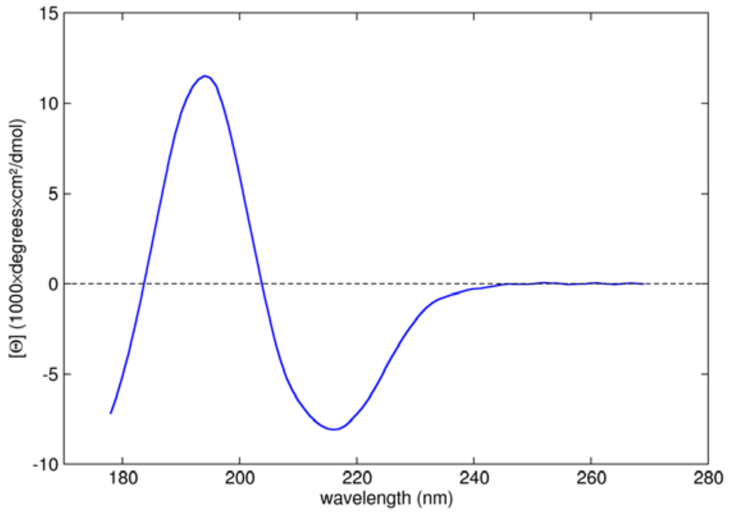

Figure ~~S4~~S3: Predicted circular dichroism spectrum of mOBP5 using SESCA software.

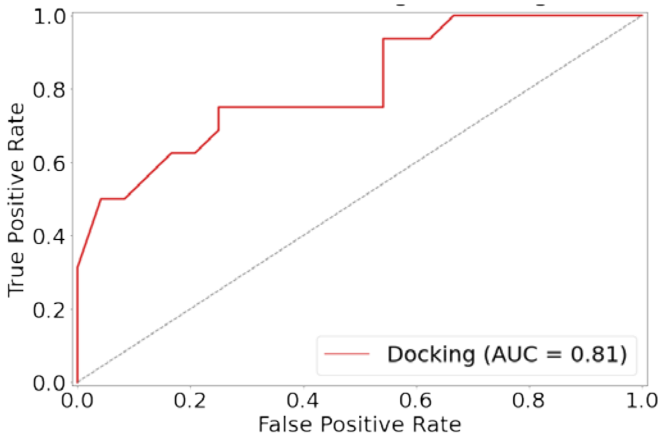

Figure ~~S5~~S4: Receiver Operating Characteristic curve (ROC), characterizing the protocols' efficacy using the binding energy score from Autodock Vina. It depicts the True positive rate (sensitivity) versus false positive rate (1 - specificity). AUC corresponds to the area under the curve. The closer the AUC gets to 1 the better the protocol is at discriminating binders and non-binders.

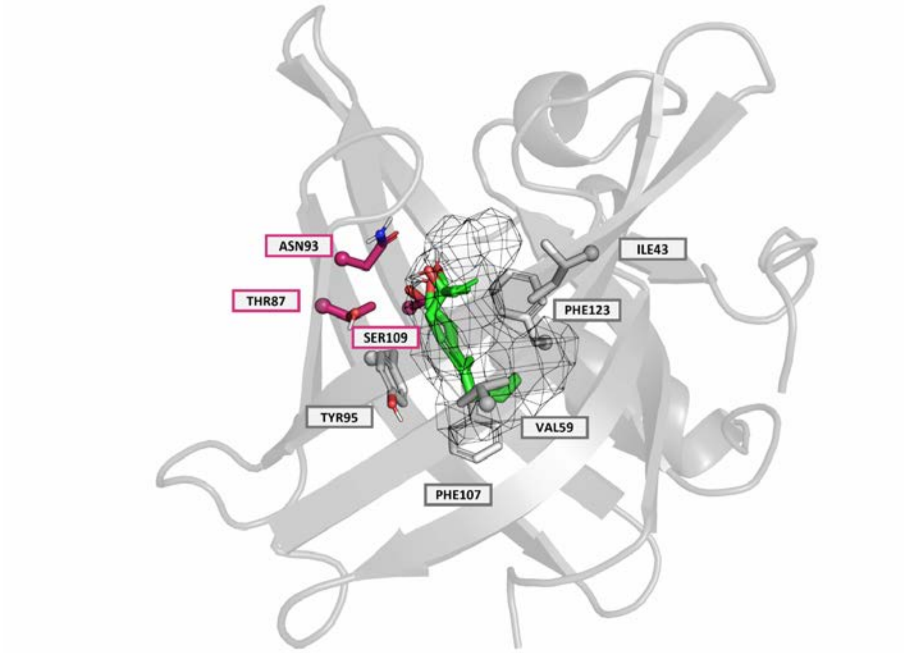

Figure ~~S6~~S5: Binding pocket residues of mOBP5, polar and apolar residues are coloured in purple and grey respectively. For each amino acid, only the side chain is represented, and the carbon alpha appears as a sphere. Each amino acids are depicted with a 3-letter code and their position in the sequence. Predicted mOBP5 internal cavity is represented as a black mesh grid. Heptanol, Octanol and Geraniol with their best predicted pose are represented in green.

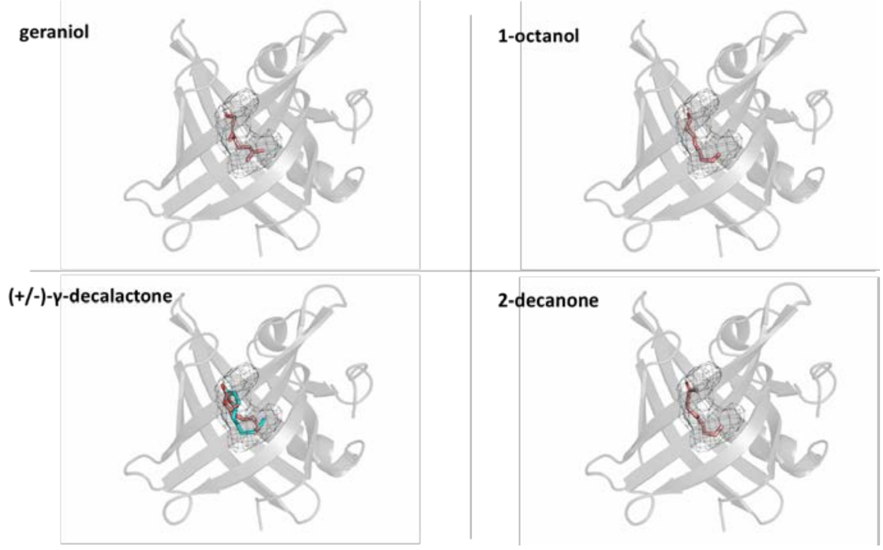

Figure ~~S7~~S6: Representation of the best pose found through docking experiments of 4 molecules (geraniol, octanol, (+/-)-γ-decalactone and 2-decanone). Odorants are represented in orange and the calyx as blue mesh grid. The two γ-decalactone enantiomers are superimposed and show a similar binding pose.

Table S1: IC_{50} values and affinity constants for the odorants used for mOBP5. For very weak ligands, if an IC_{50} value could not be extrapolated, an arbitrary value of 200 μM was assigned.

	IC_{50} (μM) [Fluo a]	K_d (μM) [Fluo b]	N [ITC c]	K_a (ITC) [ITC d]	~~1/K_a~~ K_d (μM) [ITC e]
hexanoic acid	200.00	≥3.16			
octanoic acid	200.00	≥3.16			
1-hexanol	40.0	2.60			
1-heptanol	2.4	0.59			
1-octanol	1.1	0.29	0.71	3.6E+07	0.028
thymol	40.0	2.60			
geraniol	0.7	0.19	0.71	2.5E+07	0.040
linalool	1.1	0.30			
menthol	1.5	0.38			
hexanal	200.0	≥3.16			
benzaldehyde	200.0	≥3.16			
4-methoxy-benzaldehyde	40.0	2.60			
isovanillin	200.0	≥3.16			
vanillin	200.0	≥3.16			
octanal	1.3	0.34			
cinnamal	200.0	≥3.16			
citral	2.5	0.61	0.65	1.3E+07	0.076
lilialdehyde	1.4	0.38			
pyrazine	200.0	≥3.16			
2-methyl-pyrazine	200.0	≥3.16			
2-ethyl-pyrazine	200.0	≥3.16			
3-iso-butyl-2-methoxy-pyrazine (IBMP)	7.2	1.30			
maltol	200.0	≥3.16			
cyclohexanone	200.0	≥3.16			
(-)-carvone	1.1	0.28			
(+)-carvone	1.0	0.28	0.47	8.7E+06	0.115
camphor	10.4	1.60			
menthone	0.8	0.23	0.58	1.8E+07	0.055
2-decanone	1.2	0.31			
β-ionone	3.0	0.71			
ethyl acetate	200.0	≥3.16			
ethyl butyrate	40.0	2.60			
isoamyl acetate	2.5	0.60	0.87	8.6E+06	0.116
anisole	200.0	≥3.16			
cineole	40.0	2.60			
γ-decalactone	2.4	0.58			
α-pinene	0.9	0.24			
eugenol	200.0	≥3.16			
dimethyl disulfide	200.0	≥3.16			
benzothiazole	200.0	≥3.16			

[a] IC_{50} (µM) ^{Fluo} : concentrations of the competitor ligand that caused a decrease of the NPN fluorescence of half-maximal intensity; [b] K_d (µM) ^{Fluo} : dissociation constant determined by fluorescence ; [c] N ^{ITC} : binding stoichiometry per monomer determined by ITC ; [d] K_a (ITC) ^{ITC} : association constant determined by ITC ; [e] K_d (µM) ^{ITC} : dissociation constant determined by ITC.

Table S2: Volume values and polarity score according to fpocket.

Name	PDB code	Volume (Å3)	Polarity_Score
Panda OBP3	5NGH	486	3
Pig OBP	1A3Y	502	5
Human OBPIIa	4RUN	931	11
Bovine OBP	1OBP	457	5
Rat OBP3	3ZQ3	299	3
Rat OBP1	3FIQ	401	4
Mouse OBP5	-	437	4

Formatted: Superscript
Formatted: English (United States)
Formatted: Font: (Default) Calibri, Not Superscript/ Subscript
Formatted: English (United States)
Formatted: Font: (Default) Calibri
Formatted: English (United States)
Formatted: Font: (Default) Calibri, Not Superscript/ Subscript
Formatted: Font: (Default) Calibri
Formatted: English (United States)
Formatted: English (United States), Not Superscript/ Subscript
Formatted: English (United States)
Formatted: Superscript
Formatted: English (United States)
Formatted: Superscript
Formatted: English (United States)
Formatted: Font: (Default) Calibri
